# Bombesin receptor subtype‐3‐expressing neurons regulate energy homeostasis through a novel neuronal pathway in the hypothalamus

**DOI:** 10.1002/brb3.881

**Published:** 2017-12-15

**Authors:** Minoru Maruyama, Natsu Hotta, Yasunori Nio, Kenichi Hamagami, Toshimi Nagi, Masaaki Funata, Junichi Sakamoto, Masanori Nakakariya, Nobuyuki Amano, Mayumi Nishida, Tomohiro Okawa, Yasuyoshi Arikawa, Shinobu Sasaki, Shizuo Kasai, Yasutaka Nagisa, Yugo Habata, Masaaki Mori

**Affiliations:** ^1^ Cardiovascular and Metabolic Drug Discovery Unit Takeda Pharmaceutical Company Limited Kanagawa Japan; ^2^ Extra Value Generation & General Medicine Drug Discovery Unit Takeda Pharmaceutical Company Limited Kanagawa Japan; ^3^ Central Nervous System Drug Discovery Unit Takeda Pharmaceutical Company Limited Kanagawa Japan; ^4^ Biomolecular Research Laboratories Takeda Pharmaceutical Company Limited Kanagawa Japan; ^5^ Drug Metabolism and Pharmacokinetics Research Laboratories Takeda Pharmaceutical Company Limited Kanagawa Japan; ^6^ Integrated Technology Research Laboratories Takeda Pharmaceutical Company Limited Kanagawa Japan; ^7^ Medicinal Chemistry Research Laboratories Pharmaceutical Research Division Takeda Pharmaceutical Company Limited Kanagawa Japan; ^8^ Present address: CVM Marketing Japan Pharma Business Unit Takeda Pharmaceutical Co. Ltd. 12‐10, Nihonbashi 2‐Chome, Chuo‐ku Tokyo 103‐8686 Japan; ^9^ Present address: Foods & Nutrients Yamanashi Gakuin Junior College Sakaori 2‐4‐5, Kofu Yamanashi 400‐8575 Japan

**Keywords:** bombesin receptor subtype‐3, energy homeostasis, hypothalamus, neuronal pathway, obesity

## Abstract

**Objectives:**

Bombesin receptor subtype‐3 (BRS‐3) has been suggested to play a potential role in energy homeostasis. However, the physiological mechanism of BRS‐3 on energy homeostasis remains unknown. Thus, we investigated the BRS‐3‐mediated neuronal pathway involved in food intake and energy expenditure.

**Materials and Methods:**

Expression of BRS‐3 in the rat brain was histologically examined. The BRS‐3 neurons activated by refeeding‐induced satiety or a BRS‐3 agonist were identified by c‐Fos immunostaining. We also analyzed expression changes in feeding‐relating peptides in the brain of fasted rats administered with the BRS‐3 agonist.

**Results:**

In the paraventricular hypothalamic nucleus (PVH), dorsomedial hypothalamic nucleus (DMH), and medial preoptic area (MPA), strong c‐Fos induction was observed in the BRS‐3 neurons especially in PVH after refeeding. However, the BRS‐3 neurons in the PVH did not express feeding‐regulating peptides, while the BRS‐3 agonist administration induced c‐Fos expression in the DMH and MPA, which were not refeeding‐sensitive, as well as in the PVH. The BRS‐3 agonist administration changed the *Pomc* and *Cart* mRNA level in several brain regions of fasted rats.

**Conclusion:**

These results suggest that BRS‐3 neurons in the PVH are a novel functional subdivision in the PVH that regulates feeding behavior. As the MPA and DMH are reportedly involved in thermoregulation and energy metabolism, the BRS‐3 neurons in the MPA/DMH might mediate the energy expenditure control. POMC and CART may contribute to BRS‐3 neuron‐mediated energy homeostasis regulation. In summary, BRS‐3‐expressing neurons could regulate energy homeostasis through a novel neuronal pathway.

## 1 INTRODUCTION

The central nervous system (CNS) plays an important role in energy homeostasis. The disruption of this system leads to the dysregulation of feeding behavior and energy metabolism, which is associated with obesity. Obesity contributes to the development of a large number of diseases, including diabetes mellitus, hypertension, coronary heart disease, and certain types of cancer and hence remains an important social problem (Guh et al., 2009; Pi‐Sunyer, 2009). Although several anti‐obesity agents are in the market, their efficacy is limited (Butsch, 2015). Therefore, research on CNS‐acting drugs with regulatory effects on both feeding behavior and peripheral energy metabolism could represent a potential strategy for developing potent anti‐obesity drugs.

Bombesin‐like peptides are known to serve as neuromodulators in the CNS. Bombesin was originally isolated from the skin of frogs as a 14‐amino acid peptide with smooth muscle contraction activity; thereafter, several bombesin‐like peptides were identified from various species. There are two such peptides in mammals, gastrin‐releasing peptide (GRP) and neuromedin B (NMB), which bind to the G‐protein‐coupled receptors (GPCR) GRP receptor (GRPR) and NMB receptor (NMBR), respectively (Corjay et al., 1991; Ramos‐Álvarez et al., 2015; Whitley, Moore, Giraud, & Shulkes, 1999). Bombesin receptor subtype‐3 (BRS‐3) was identified as the third member of this GPCR subfamily, based on sequence similarity, and was found to be expressed in the CNS (Fathi et al., 1993). However, despite the sequence similarity, BRS‐3 does not have a high affinity with any known bombesin‐like peptides, and its natural ligand remains unknown (Ramos‐Álvarez et al., 2015). Although the physiological function of BRS‐3 remains unclear, BRS‐3‐deficient (Brs‐3^‐/Y^) mice are known to develop obesity and have a reduced metabolic rate and increased food intake (FI) (Guan et al., 2010; Ladenheim et al., 2008; Ohki‐Hamazaki et al., 1997; Yamada, Santo‐Yamada, Wada, & Wada, 2002). Several nonpeptide BRS‐3 agonists with an anti‐obesity effect in animals have recently been developed (Chobanian et al., 2012; Guan et al., 2010, 2011; Lateef, Abreu‐Vieira, Xiao, & Reitman, 2014; Matsufuji et al., 2015; Metzger et al., 2010; Sebhat et al., 2011). In particular, Guan et al. reported that Bag‐1, a potent and selective small‐molecule BRS‐3 agonist, increased the fasting metabolic rate and body temperature and reduced the FI and body weight (BW) in mice. These results suggest that BRS‐3 might serve as an attractive drug target for treating obesity (Xiao & Reitman, 2016).

Feeding and energy metabolism are regulated by the neural network of central and peripheral nervous systems, in which the hypothalamus plays a central role. The neurons in the hypothalamus receive inputs from the periphery through the brain stem and from the midbrain and cortex, which subsequently integrate the information associated with the energy status (Waterson & Horvath, 2015). In this neural network, the arcuate nucleus (ARC), paraventricular hypothalamic nucleus (PVH), ventromedial nucleus of the hypothalamus, and dorsomedial nucleus of the hypothalamus (DMH) play important roles with the help of multiple hypothalamic peptides (van Swieten, Pandit, Adan, & van der Plasse, 2014; Waterson & Horvath, 2015). For instance, neuropeptide Y (NPY), agouti‐related peptide (AgRP), orexin, and melanin‐concentrating hormone (MCH) induce hyperphagia, whereas α‐melanocyte‐stimulating hormone (α‐MSH), oxytocin, corticotropin‐releasing hormone (CRH), and arginine vasopressin (AVP) induce anorexia (Crespo, Cachero, Jiménez, Barrios, & Ferreiro, 2014). These peptides are also involved in the control of peripheral energy expenditure (EE) through the sympathetic nervous system. Furthermore, the preoptic area is known to represent the center of autonomic thermoregulation and is involved in peripheral EE via the DMH and brainstem (Morrison, 2016). As BRS‐3 is reportedly expressed in the hypothalamus (Jennings et al., 2003; Liu et al., 2002; Sano et al., 2004; Zhang et al., 2013), it is expected to regulate energy homeostasis through this hypothalamic neural network. Nevertheless, the precise neural pathway underlying the BRS‐3 agonist‐mediated anti‐obesity effect remains unclear.

Recently, we reported a novel selective nonpeptide BRS‐3 agonist, compound‐A (Nio et al., 2017). Compound‐A shows clear anorectic effects and enhanced energy expenditure in rats, suggesting that this compound is an useful tool compound to assess the function of BRS‐3 neurons. Thus, in this study, we examined the hypothalamic neural network underlying the BRS‐3‐expressing neuron‐mediated energy homeostasis regulation using compound‐A.

## 2 MATERIALS AND METHODS

### 2.1 Compounds

Compound‐A (active conformer (tR2(IC)), 6‐benzyl‐*N*‐*tert*‐butyl‐9‐methoxy‐7‐oxo‐6,7‐dihydro‐*5H*‐dibenzo[c,e]azepine‐5‐carboxamide) and compound‐C (racemate) were synthesized at Takeda Pharmaceutical Company Limited as previously reported (Nio et al., 2017). Sibutramine and CL316,243 were purchased from Wako Pure Chemical (Osaka, Japan) and Tocris Bioscience (Bristol, UK), respectively. The compounds were suspended in 0.5% methylcellulose solution for oral dosing.

### 2.2 Animals

F344/Jcl rats (male, 5‐week‐old) were purchased from CLEA Japan (Tokyo, Japan). They were fed on a high‐fat diet (HFD; D12451: Research Diets, NJ, USA) from the age of 5 weeks to achieve diet‐induced obesity (DIO). Male Sprague Dawley (SD) rats were also purchased from CLEA Japan. Melanin‐concentrating hormone receptor‐1‐deficient (Mchr1^−/−^) mice were originally established through the targeted disruption of exon 2 of the *Mchr1* gene and backcrossed to a C57BL/6J background for four times with a speed congenic system. All animals were maintained at an appropriate temperature (23–25°C) under a 12‐hr light and dark cycle (7:00–19:00 for rats, 7:30–19:30 for Mchr‐1^−/−^ mice). All the animal experiments were conducted in compliance with a protocol that was reviewed by the Institutional Animal Care and Use Committee of Takeda Pharmaceutical Company Limited.

### 2.3 In vitro agonistic activity

With regard to functional assays, the agonist‐induced mobilization of intracellular Ca^2+^ was measured in CHO‐K1 cells that overexpressed BRS‐3 using an aequorin bioluminescence assay (duplicate experiments).

### 2.4 Pharmacokinetic parameters of compound‐A in SD rats

To determine the pharmacokinetic parameters of compound‐A, male 8‐week‐old SD rats (*n* = 3) were orally administered certain compounds (1 mg/kg BW/5 ml, suspended in 0.5% methylcellulose aqueous solution) via cassette dosing. The plasma compound concentrations were measured using liquid chromatography–tandem mass spectrometry.

### 2.5 Measurement of FI and BW after compound‐A administration in SD or DIO‐F344 rats

Male SD rats (10‐week‐old) were divided into five groups (4–5 rats in each group) based on the nocturnal FI during the previous night and the BW on the morning of the drug administration. The SD rats were orally administered with vehicle (0.5% methylcellulose solution), compound‐C (3, 10, and 30 mg/kg), or sibutramine (10 mg/kg) at 1–2 hr prior to the onset of the dark phase. FI was measured 24 hr after drug administration, whereas BW was measured 24 hr after drug administration. Two weeks before the study, DIO‐F344 rats were fed on a powdery HFD and had been acclimatized to oral dosing. The DIO‐F344 rats (male, 47‐, 52‐ or 53‐week‐old) were divided into four or five groups (4–8 rats in each group) based on the nocturnal FI during the previous night and the BW on the morning of the drug administration. The DIO‐F344 rats were orally administered with vehicle (0.5% methylcellulose solution), compound‐A or compound‐C (3, 10, and 30 mg/kg), or sibutramine (1 mg/kg) at 1–2 hr prior to the onset of the dark phase. FI was measured 4, 16, and 24 hr after drug administration, whereas BW was measured 24 hr after drug administration.

### 2.6 Measurement of EE after compound‐A administration in fasted DIO‐F344 rats

DIO‐F344 rats (male, 57‐week‐old) underwent fasting for 16 hr and were then housed individually in the metabolic chamber of an Oxymax system (Columbus Instructions, OH, USA) for acclimatization to the chamber. The rats were divided into three groups according to the BW and EE at approximately 10 a.m. for 1 hr. At 11:00 a.m., the rats were orally administered with vehicle (0.5% methylcellulose solution), compound‐A (30 mg/kg), or CL316,243 (2 mg/kg), and the heat production and respiratory exchange ratio (RER) were measured over 5 hr with 10‐min interval. Data were averaged in every 30 min.

### 2.7 Effect of compound‐A on FI and BW in Mchr‐1^−/−^ mice

Male Mchr‐1^−/−^ mice and age‐matched male wild‐type mice (38‐week‐old) were individually housed and fed on a HFD (D‐12451) from 6 weeks of age and were acclimatized to oral dosing for 1 week. They were divided into three groups according to the nocturnal FI of the previous day and the BW on the morning of the day of drug administration. They were orally administered vehicle (0.5% methylcellulose solution), compound‐A (100 mg/kg), or sibutramine (10 mg/kg). The FI and BW were measured at 16 and 24 hr after drug administration, respectively.

### 2.8 Histochemistry

To determine the expression of BRS‐3 mRNA and feeding‐related neuropeptides, brain samples from adult male SD rats (7‐week‐old) were examined. To identify the c‐Fos‐immunoreactive (ir) neurons after refeeding, brain samples were collected from adult male SD rats (11‐week‐old) following 48‐hr fasting or 2‐hr refeeding after 48‐hr fasting. To indicate the c‐Fos‐ir neurons following compound‐A administration, the DIO‐F344 rats (male, 43‐week‐old) were orally administered vehicle (0.5% methylcellulose solution) or compound‐A (30 mg/kg) at 9:00 a.m. in an ad libitum condition, and the brains were sampled 2 hr after dosing.

For single c‐Fos immunohistochemistry (IHC), single in situ hybridization (ISH), and double staining for ISH and IHC, the animals were anesthetized and perfused with saline, followed by 4% paraformaldehyde, via the left cardiac ventricle, prior to brain sampling. After the brains were cryoprotected with 30% sucrose, frozen coronal sections (40 μm) were prepared with a freezing cryostat and used for staining. For double ISH, the animals were anesthetized, and the removed brains were frozen using O.C.T. compound (Sakura Finetek Japan, Tokyo, Japan) in liquid nitrogen. Coronal fresh‐frozen sections were cut to 16 μm and used for staining.

For ISH, the cDNA fragments of the *Brs3* (NM_152845: 121‐1320), *Crh* (NM_031019: 176‐739), *Npy* (NM_012614: 126‐527), and *Pomc* (NM_139326: 75‐760) were obtained by polymerase chain reaction (PCR) and were subcloned into the pCR‐BluntII‐TOPO vector (Invitrogen, K280020, CA, USA). Digoxygenin (DIG)‐ and fluorescein (FITC)‐labeled riboprobes were produced from these plasmids as templates via in vitro transcription.

For single IHC of c‐Fos, free‐floating coronal sections (40 μm) were incubated with anti‐c‐Fos antibody (Santa Cruz Biotechnology, sc‐52; 1/4000, CA, USA: RRID AB_2106783) (Table 1) and then visualized using the VECTASTAIN Elite ABC Kit (Vector Laboratories, PK‐6101, CA, USA) and diamino‐benzidine. All the procedures were performed with the free‐floating method.

**Table 1:** List of primary antibodies

Antigen	Description of immunogen	Source, host species, cat. #, RRID	Dilution	References
Fos	Peptide corresponding to residues 3‐16 of human c‐Fos	Santa Cruz Biotechnology, rabbit polyclonal, sc‐52, AB_2106783	1/4000	Brown et al. (1998)
Fos	Peptide corresponding to residues 128–152 of human c‐Fos	Santa Cruz Biotechnology, rabbit polyclonal, SC‐253, AB_2231996	1/4000	Konsman & Blomqvist (2005)
Oxytocin	Rat oxytocin	Calbiochem, rabbit polyclonal, PC226L, AB_2157630	1/400	Luo et al. (2002)
AVP	[Arg^8^]‐Vasopressin	Phoenix, rabbit polyclonal, H‐065‐07, AB_2715552	1/2000	Yi et al. (2008)

For single ISH of BRS‐3, free‐floating coronal sections (40 μm) were treated with proteinase K, acetylated, and then incubated in a hybridization buffer containing DIG‐labeled riboprobes at 60°C. The sections were washed, treated with alkaline phosphatase‐conjugated anti‐DIG antibody (Roche, 1093274; 1/1000, IN, USA: RRID AB_514497), and then visualized with 4‐nitroblue tetrazolium chloride (NBT) and 5‐bromo‐4‐chloro‐3‐indolyl phosphate (BCIP). For double staining of ISH (BRS‐3) and IHC (oxytocin or AVP), the sections obtained after ISH were incubated with rabbit anti‐oxytocin antibody (Calbiochem, PC226L; 1/400, CA, USA: RRID AB_2157630) or rabbit anti‐AVP antibody (Phoenix, H‐065‐07; 1/2000, CA, USA: RRID: AB_2715552) (Table 1) and then visualized using goat anti‐rabbit IgG Alexa 488 (Invitrogen, A11034; 10 μg/ml: RRID AB_2576217). The ISH signals were converted to red under the imaging software. For double staining with ISH (BRS‐3) and IHC (c‐Fos), the sections obtained after ISH were incubated with rabbit anti‐c‐Fos antibody (Santa Cruz, SC‐253; 1/4000: RRID AB_2231996) (Table 1) and then visualized using the VECTASTAIN Elite ABC Kit and diamino‐benzidine. All the procedures were performed with the free‐floating method.

For double ISH of BRS‐3 and CRH, NPY, or proopiomelanocortin (POMC), coronal fresh‐frozen sections on a glass slide (16 μm) were fixed with 4% paraformaldehyde. The fixed sections were acetylated and then incubated in a hybridization buffer containing DIG (*Brs3*)‐ and FITC (*Crh*,* Npy*, and *Pomc*)‐labeled riboprobes at 60°C. The sections were washed, treated with phosphatase‐conjugated anti‐DIG antibody, and then visualized using NBT and BCIP, wherein the color was converted to red under an imaging software. To detect the FITC probe, the sections were incubated with anti‐FITC antibody HRP conjugate (PerkinElmer, NEF710; 1/500, MA, USA: RRID AB_2314403) and then treated with CSA II (DAKO Japan, K149711, Kyoto, Japan) to enhance the FITC signal.

After processing, the sections were mounted and then examined via light microscopy or confocal laser microscopy.

### 2.9 Antibodies

Primary antibodies used in this study are listed in Table 1. We used two different anti‐c‐Fos antibodies, Santa Cruz Biotechnology sc‐52 and sc‐253. sc‐52 recognized the predicted molecular weight of 62 kDa on Western blots (manufacturer's datasheet), and it has been validated for detection of nuclear‐localized c‐Fos proteins (Brown, Gentry, & Rowland, 1998). sc‐253 was used for immunostaining after ISH reaction. This antibody recognized the predicted molecular weight of 62 kDa on Western blots (manufacturer's datasheet) and it has been validated for detection of nuclear‐localized c‐Fos proteins (Konsman & Blomqvist, 2005). For the marker of oxytocin‐containing neuron, anti‐oxytocin antibody (Calbiochem, PC226L) was used. Staining is completely eliminated by pretreatment of antibody with oxytocin, but no staining is detected by pre‐absorption of antibody with vasopressin (manufacturer's datasheet). This antibody has been validated for detection of cell bodies and fibers in oxytocin mRNA localized nucleus, such as PVH and supraoptic nucleus (Luo, Kaur, & Ling, 2002). For the marker of AVP‐containing neuron, anti‐AVP antibody (Phoenix, H‐065‐07) was used. This antibody does not react with oxytocin (manufacturer's datasheet) and it has been validated for detection of cell bodies and fibers in AVP‐mRNA localized nucleus, such as PVH and supraoptic nucleus (Yi et al., 2008).

### 2.10 Quantitative analysis of the images

To avoid overcounting, evenly spaced series of sections (80‐ or 120‐μm interval) including regions of interest (ROIs) were prepared and stained. The sections for PVH, DMH, medial preoptic area (MPA), ARC, and NTS were dissected from the areas including bregma −1.0 to −2.3, −2.2 to −3.5, −0.2 to −1.0, −1.8 to −4.2, and −13.7 to −14.3 mm, respectively (Paxinos & Watason, 2004). After sections at both end of ROI were removed, two to four sections in each animal were selected and the 0.58‐ or 0.29‐mm^2^ images, including the unilateral nucleus, were acquired using Nikon ECLIPSE E800 (two images were obtained from one section). The ROIs were determined according to the positional relationship with the surrounding brain structures such as fornix and 3rd ventricle (PVH and DMH), anterior commissure and 3rd ventricle (MPA), 3rd ventricle (ARC), and area postrema and central canal (NTS) (Paxinos & Watason, 2004). Images including ROI were acquired, and the all signals in the images were analyzed. The number of c‐Fos‐positive cells was automatically measured from the 0.58‐mm^2^ images with Image‐Pro Plus (MediaCybernetics, MD, USA). The counts from two or three sections (four to six images) in each animal were analyzed, and the average density of c‐Fos‐positive cells in 0.58‐mm^2^ area of each animal was calculated. The value was used for the subsequent statistical analysis. Average number of total c‐Fos‐positive cells in each animal is shown in figure legends. The percentages of c‐Fos‐positive cells in the BRS‐3 mRNA‐positive neurons were calculated from the acquired 0.29‐mm^2^ images; two to four sections (four to eight images) in each animal were analyzed and total number of BRS‐3 mRNA‐positive cells and BRS‐3 mRNA/c‐Fos double‐positive cells in ROI were manually counted. Then, the percentage of c‐Fos‐positive cells in the BRS‐3 mRNA‐positive neurons in each animal was calculated (% of c‐Fos/BRS‐3 double‐positive cells in ROI). The value was used for the subsequent statistical analysis. Average number of the analyzed BRS‐3 mRNA‐positive cells in each animal is shown in figure legends.

### 2.11 Quantitative reverse transcriptase (RT)‐PCR of peptides in the brain regions of DIO‐F344 rats after compound‐A administration

DIO‐F344 rats (male, 53‐week‐old) were orally administered vehicle (0.5% methylcellulose solution) or compound‐A (30 mg/kg) at 9:00 a.m. following fasting for 16 hr or under free access food and water conditions. At 1 hr after dosing, the rats were anesthetized, and the samples including ARC, PVH, lateral hypothalamic area (LHA), or nucleus tractus solitarius (NTS) were dissected. In detail, 1 mm of brain slices was prepared using Brain Matrix (Muromachi Kicai, BS‐Z 2000C, Tokyo, Japan), and then, the area including ROI was roughly dissected by razors. Total RNA was extracted using ISOGEN (Nippon Gene, Tokyo, Japan), and then, cDNA was prepared from total RNA by oligo (dT) primer using SuperScript II reverse transcriptase (Invitrogen). Expression analysis (TaqMan RT‐PCR) was performed by ABI Prism 7900HT (Applied Biosystems, MA, USA) using TaqMan^®^ Gene Expression Master Mix (Applied Biosystems), with the primers and probes listed in Table 2. For rat cyclophilin, predesigned primers and probe (Rn00452692_m1; Assays‐on‐Demand, Applied Biosystems) were used. The mRNA expression in each gene was normalized to the cyclophilin mRNA expression using ddCT method.

**Table 2:** TaqMan probe and primer sequences used for the expression analysis

Gene name	Probe/Primer	Sequence	5′	3′
*Pomc*	Probe	CAGGCCCGGATGCAAGCCAGCA	FAM	TAMRA
	Forward primer	CATAGACGTGTGGAGCTGGTG		
	Reverse primer	CCGCCGAGAGGTCGAGTC		
*Cart*	Probe	CCGCCTTGGCAGCTCCTTCTCATGG	FAM	TAMRA
	Forward primer	ATCTACTCTGCCGTGGATGATG		
	Reverse primer	GCGCTTCAATCTGCAACACA		
*Npy*	Probe	CCCAGAACAAGGCTTGAAGACCCTTCCATG	FAM	TAMRA
	Forward primer	GAGACACTGATTTCAGATCTCTTAATGAG		
	Reverse primer	GTCAGGAGAGCAAGTTTCATTTCC		
*Agrp*	Probe	CGGTTCTGTGGATCTAGCACCTCTGCC	FAM	TAMRA
	Forward primer	GCAGCAGACCGAGCAGAAG		
	Reverse primer	CACAGCGACGCGGAGAAC		
*Hcrt*	Probe	CCTTCCTTCTACAAAGGTTCCCTGGGCC	FAM	TAMRA
	Forward primer	GGATTGCCTCTCCCTGAGC		
	Reverse primer	AGCAGCAGCAGCGTCAC		
*Pmch*	Probe	ACAAGACCACAAAGAACACAGGCTCCAAG	FAM	TAMRA
	Forward primer	AAAATGATGAGAGCGGCTTCATG		
	Reverse primer	GCAGACCGTGAGTTACGAGATT		
*Crh*	Probe	CGCTCTCTTCTCCTCCCTTGGCAGGT	FAM	TAMRA
	Forward primer	ACCTCGCAGAACAACAGTGC		
	Reverse primer	CCGCAGCCGCATGTTTAGG		
*Oxt*	Probe	CGGATGGCTGCCGCACCGACC	FAM	TAMRA
	Forward primer	CGCGGGCATCTGCTGTAG		
	Reverse primer	GCCCTAAAGGTATCATCACAAAGC		
*Avp*	Probe	CCACATCCGACATGGAGCTGAGACAGTGT	FAM	TAMRA
	Forward primer	CTACTTCCAGAACTGCCCAAGAG		
	Reverse primer	GAAGCAGCGCCCTTTGCC		

### 2.12 Statistical analysis

Data involving more than two groups were assessed by one‐way analysis of variance (ANOVA) followed by Williams test. Differences between two groups were assessed using Student's *t* test or Aspin–Welch's *t* test. In Figure 2, statistical differences were analyzed with Student's *t* test or Aspin–Welch test, followed by Bonferroni's correction, for 9‐time point comparisons.

## 3 RESULTS

### 3.1 Profile of the BRS‐3 agonist compounds

Compound‐A is an active conformer (tR2(IC)) of BRS‐3 agonist as previously reported (Nio et al., 2017). Compound‐A had agonistic activity with an EC_50_ value of 100 nM (95% confidence interval: 59–172 nM) as per the aequorin assay (Ca^2+^) against rat BRS‐3, but did not show agonistic action at 10 uM to human GRPR and NMBR. Compound‐C is the racemate of compound‐A (Nio et al., 2017) and had agonistic activity with an EC_50_ value of 130 nM against rat BRS‐3 (Ca^2+^). The pharmacokinetic profile of compound‐A (1 mg/kg, po) in SD rats was determined and the maximum plasma concentration (Cmax), time at which the Cmax was observed (Tmax), and bioavailability (BA) were found to be 69.1 ng/ml, 0.5 hr, and 21.7%, respectively. Our previous study revealed that the compound‐C can pass the blood–brain barrier, suggesting that compound‐A could pass the blood–brain barrier (Nio et al., 2017).

### 3.2 Anti‐obesity effect of single oral administration of compound‐A in DIO‐F344 rats

**Figure 1:**
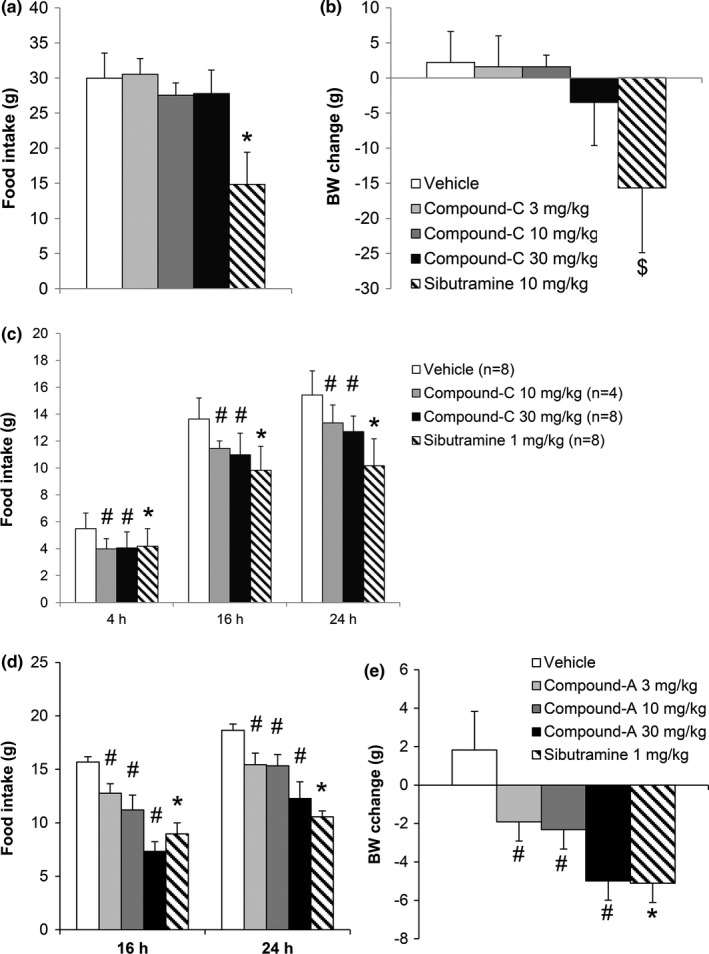
Suppression of food intake and body weight by compound‐A in normal chow‐fed SD rats or DIO‐F344 rats. (a, b) Food intake at 24 hr (a) and body weight change at 24 hr (b) after single oral administration of compound‐C (3, 10, and 30 mg/kg) and sibutramine (10 mg/kg) in normal chow‐fed SD rats. (c) Food intake at 4, 16, and 24 hr (c) after single oral administration of compound‐C (3, 10, and 30 mg/kg) and sibutramine (1 mg/kg) in DIO‐F344 rats. Results are presented as mean values ± standard deviation (*n* = 4–8). (d, e) Food intake at 16 and 24 hr (d) and body weight change at 24 hr (e) after single oral administration of compound‐A (3, 10, and 30 mg/kg) and sibutramine (1 mg/kg). Results are presented as mean values ± standard deviation (*n* = 6). #*p* < .025 versus vehicle (Williams test), *p < .05 versus vehicle (Student's *t* test), ^$^ *p* < .05 versus vehicle (Aspin–Welch test)

We examined the effect of compound‐A and compound‐C on the FI and BW of SD or DIO‐F344 rats. Single oral administration of compound‐C (3, 10, and 30 mg/kg) did not significantly decrease the FI and BW at 24 hr in normal chow‐fed SD rats (Figure 1a and b) but significantly decreased the FI in a dose‐dependent manner at 4, 16, and 24 hr in DIO‐F344 rats (Figure 1c). In DIO‐F344 rats, the single oral administration of compound‐A (3, 10, and 30 mg/kg) significantly decreased the FI in a dose‐dependent manner at 16 and 24 h (Figure 1d). A significant BW reduction due to compound‐A administration at 24 hr was observed in a dose‐dependent manner (Figure 1e). The single oral administration of sibutramine (1 mg/kg), used as a positive control, also led to a decrease in FI at 16 and 24 hr and in BW at 24 hr (Figure 1d and e).

**Figure 2:**
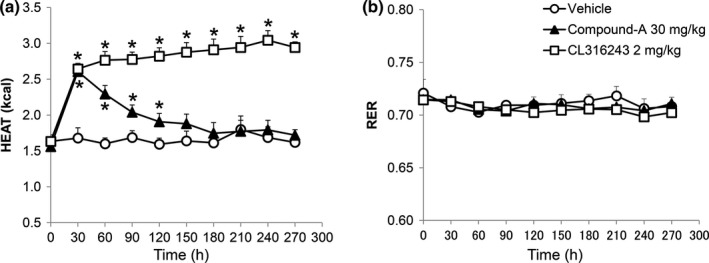
Enhancement of energy expenditure by compound‐A in fasted DIO‐F344 rats. (a, b) Heat production (kcal/hr/rat) (a) and respiratory exchange ratio (RER) (b) were measured after single oral administration of compound‐A (30 mg/kg) and CL316,243 (2 mg/kg) for 270 min. BW of vehicle, compound‐A, and CL316,243‐administered rats was 492.6 ± 10.7, 494.1 ± 22.0, and 480.7 ± 20.0 g, respectively (mean values ± standard deviation). Results are presented as mean values ± standard deviation (*n* = 5, CL316,243 2 mg/kg: *n* = 3). *p < .05 versus vehicle (Student's *t* test followed by Bonferroni's correction for 9‐time point comparisons)

Next, we examined the effects of the single administration of compound‐A on the EE in DIO‐F344 rats fasted for 16 hr. The single oral administration of compound‐A (30 mg/kg) significantly induced transient heat production without any change in the RER (Figure 2a,b). On the other hand, the β3 adrenoceptor agonist, CL316,243 (2 mg/kg), showed sustained heat production without any change in the RER (Figure 2a,b). Vehicle administration slightly increased heat production just after the administration (data not shown). BW of vehicle, compound‐A, and CL316,243‐administered rats was 492.6 ± 10.7, 494.1 ± 22.0, and 480.7 ± 20.0 g, respectively (mean values ± standard deviation).

### 3.3 Distribution of BRS‐3 mRNA in the adult rat brain

**Figure 3:**
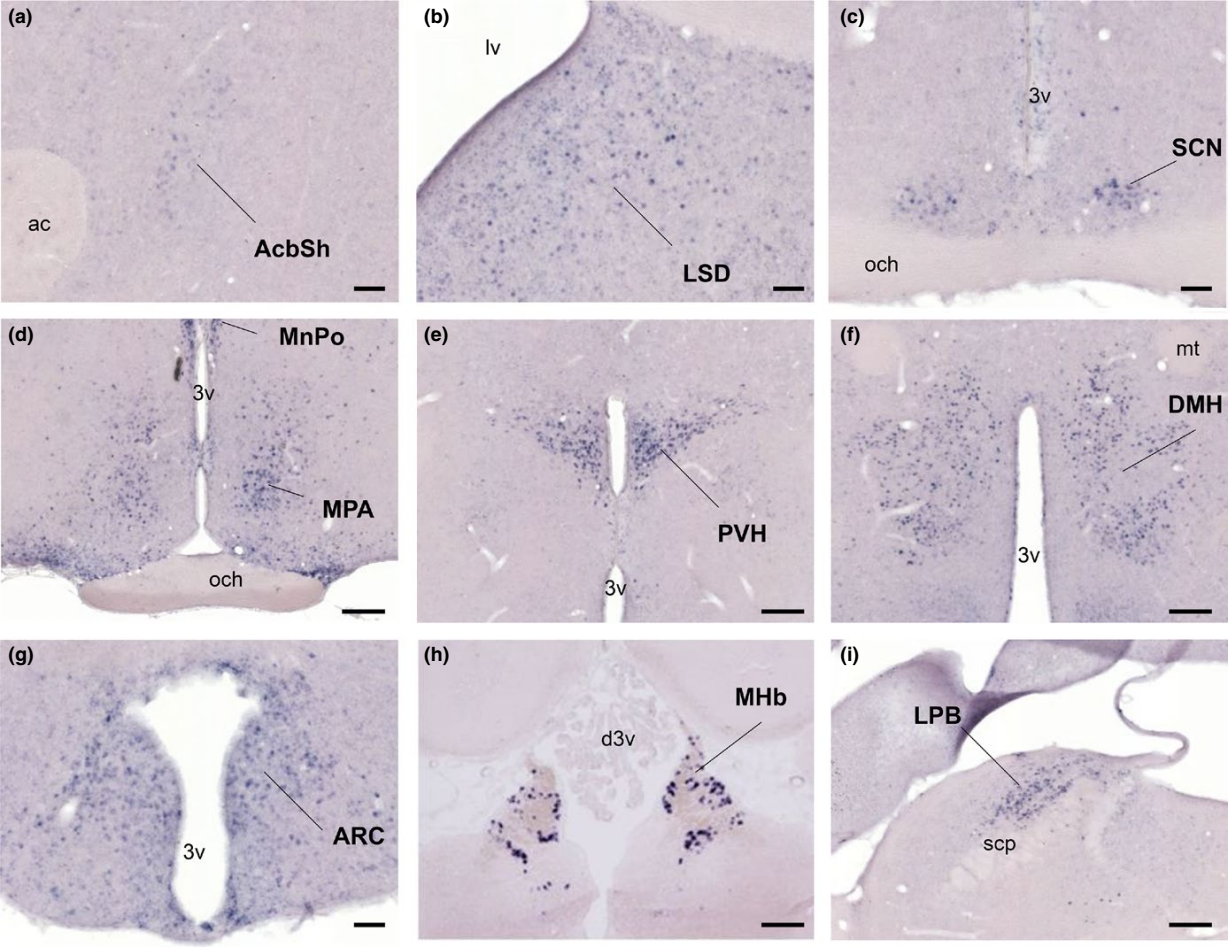
Distribution of BRS‐3 mRNA in the rat CNS. (a–i) Coronal rat brain sections were stained with a rat BRS‐3 antisense cRNA probe. Results and abbreviations are summarized in Table 3. Bar: 500 μm (a–c, g), 250 μm (d–f, h, i). 3v, 3rd ventricle; ac, anterior commissure; d3v, dorsal 3rd ventricle; mt, mammillothalamic tract; lv, lateral ventricle; och, optic chiasm; scp, superior cerebellar peduncle

**Figure 4:**
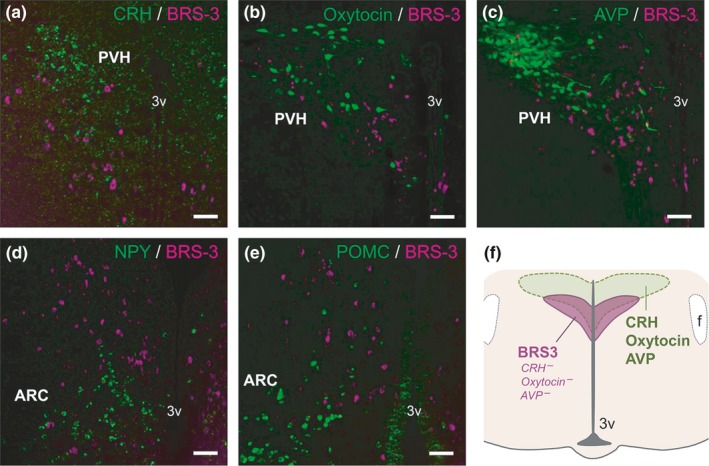
Double staining of BRS‐3 mRNA with various feeding‐related neuropeptides in the rat paraventricular hypothalamic nucleus (PVH) and ARC. (a–c) Coronal rat brain sections of the PVH showing BRS‐3 mRNA (magenta) and (a) CRH mRNA signals, and (b) oxytocin‐ir signals and (c) AVP‐ir signals (green). (d, e) Coronal rat brain sections of the ARC (bregma −3.7 mm) showing BRS‐3 mRNA (magenta) and (d) NPY mRNA signals and (e) POMC mRNA signals (green). f, Schematic representation showing the localization of the BRS‐3‐expressing neuronal cluster in the PVH. Bar: 50 μm. 3v, 3rd ventricle; f, fornix

To demonstrate the localization of BRS‐3 mRNA within the CNS, we performed an ISH study in adult SD rat brains. ISH was performed using a rat BRS‐3 cRNA probe in coronal sections and showed specific signals for the antisense probe on cell cytoplasm in a wide range of CNS regions (Figure 3). Distinct localization of signals in nuclei suggested the BRS‐3 expression in neurons. In particular, strong positive signals were detected at the MPA, median preoptic nucleus (MnPO), PVH, DMH, medial habenular nucleus (MHb), and lateral parabrachial nucleus (LPB) (Figure 3d–f,h). Moreover, moderate‐to‐low signals were observed at the accumbens nucleus shell (AcbSh), lateral septal nucleus dorsal part (LSD), suprachiasmatic nucleus (SCN), and ARC (Figure 3a–c and g). In the PVH, the signals were mainly localized at the parvicellular part (PVHp), as shown in Figures 3e and 6a. Most of the signals in the ARC were observed in the medial posterior and lateroposterior part (ArcMP and ArcLP) (Figure 3g), where the number of orexigenic NPY and anorexigenic POMC mRNA‐positive neurons was less as compared to those in the anterior part in our ISH study (Figure 4d,e). These data were similar to the results of a previous study on BRS‐3 mRNA distribution in the CNS of rats and mice (Liu et al., 2002; Zhang et al., 2013). The data are summarized in Table 3.

**Table 3:** Distribution of BRS‐3 mRNA in the rat central nervous system

Abbreviations	Structures	BRS‐3 mRNA
AcbSh	Accumbens nucleus shell	+
LSD	Lateral septal nucleus dorsal part	++
IPAC	Interstitial nucleus of the posterior limb of the anterior commissure	+
MPA	Medial preoptic area	+++
MnPO	Median preoptic nucleus	+++
VLPo	Ventrolateral preoptic nucleus	+++
SCN	Suprachiasmatic nucleus	+
PVH	Paraventricular hypothalamic nucleus	+++
DMH	Dorsomedial hypothalamic nucleus	+++
PH	Posterior hypothalamic nucleus	+++
LH	Lateral hypothalamic area	+
PeFLH	Perifornical part of lateral hypothalamus	++
ArcMP	Arcuate nucleus, medial posterior part	++
ArcLP	Arcuate nucleus, lateroposterior part	++
TMN	Tuberomammillary nucleus	+
MHb	Medial habenular nucleus	+++
MeA	Medial amygdaloid nucleus	+
CA3	Field CA3 of the hippocampus (not pyramidal cell layer)	+
LPB	Lateral parabrachial nucleus	+++
LC	Locus coeruleus	+

+++, highest; ++, moderate density; +, low density;

### 3.4 Localization of feeding‐relating neuropeptides in BRS‐3 mRNA‐positive neurons

Neurons in the PVH and ARC secrete several neuropeptides that regulate feeding behavior, such as CRH, oxytocin, AVP, NPY, and α‐MSH processed from POMC. We performed double staining of BRS‐3 mRNA with these peptides in adult SD rats to demonstrate their colocalization. Oxytocin and AVP were detected using IHC, whereas CRH, NPY, and POMC were examined using ISH. In the PVH, BRS‐3 mRNA‐positive neurons did not show any positive CRH mRNA signals (Figure 4a) nor oxytocin‐ir (Figure 4b) and AVP‐ir (Figure 4c) signals. The cluster of BRS‐3 mRNA‐positive PVH neurons in the PVH appeared to represent typical neuropeptide‐containing neurons such as CRH, oxytocin, and AVP neurons, but the location of these neurons seemed to shift slightly to the ventral portion in comparison with the typical neuropeptide‐containing neurons (Figure 4f), consistent with the findings of Zhang et al. (Zhang et al., 2013). As mentioned previously, the cluster of BRS‐3 mRNA‐positive neurons in the ARC was mainly located in the posterior part, and the neurons in the cluster were not colocalized with NPY or POMC mRNA‐positive signals (Figure 4d and e). In addition, Zhang et al. and Bagnol et al. also reported that only a few oxytocin‐ and CRH‐positive neurons and none of the TRH‐ and AVP‐positive neurons expressed BRS‐3 mRNA in the PVH and that none of the NPY‐, POMC‐, and CART‐positive neurons expressed BRS‐3 mRNA in the ARC (Bagnol & Grottick, 2008; Zhang et al., 2013). These observations suggested that most of the BRS‐3‐expressing neurons in the PVH and ARC are distinct from the neurons expressing well‐known orexigenic and anorexigenic neuropeptides.

### 3.5 Induction of c‐Fos proteins in the BRS‐3 mRNA‐positive neurons following refeeding

**Figure 5:**
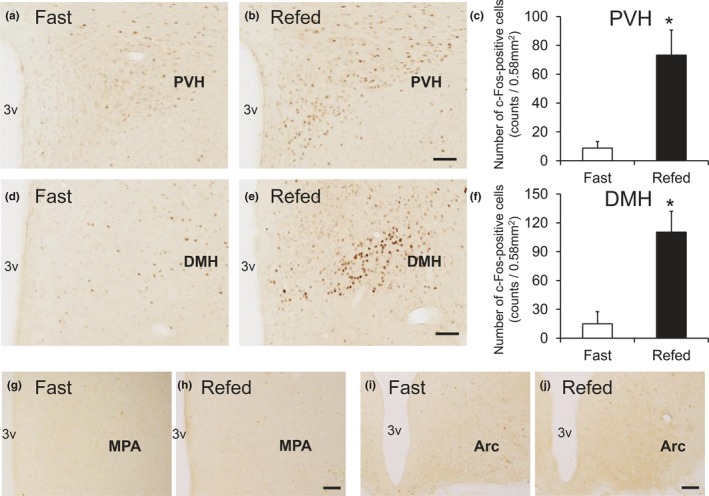
Hypothalamic c‐Fos induction in the rats refed for 2 hr after 48 hr of food deprivation. Coronal brain sections from rats fasted for 48 hr (Fast) or refed for 2 hr (Refed) were stained with anti‐c‐Fos antibody. (a–f) c‐Fos induction in the paraventricular hypothalamic nucleus (PVH) (a–c) and dorsomedial hypothalamic nucleus (DMH) (d–f) following refeeding. a, b, d, and e show representative photographs indicating the c‐Fos‐ir signals in PVH (a and b, bregma −1.9 mm) and DMH (d and e, bregma −3.2 mm). c and f show the calculated density of c‐Fos‐ir cells in the 0.58‐mm^2^ area. Average number of the total c‐Fos‐ir cells in each animal was follows; 35 ± 18 (PVH, Fast), 293 ± 70 (PVH, Refed), 60 ± 50 (DMH, Fast), and 441 ± 87 (DMH, Refed). Results are presented as mean values ± standard deviation (*n* = 3). *p < .05 versus vehicle (Student's *t* test). (g–j) Photographs showing c‐Fos‐ir signals in the MPA (g and h, bregma −0.3 mm) and ARC (i and j, bregma −3.8 mm). Bar: 100 μm. 3v, 3rd ventricle; MPA, medial preoptic area

**Figure 6:**
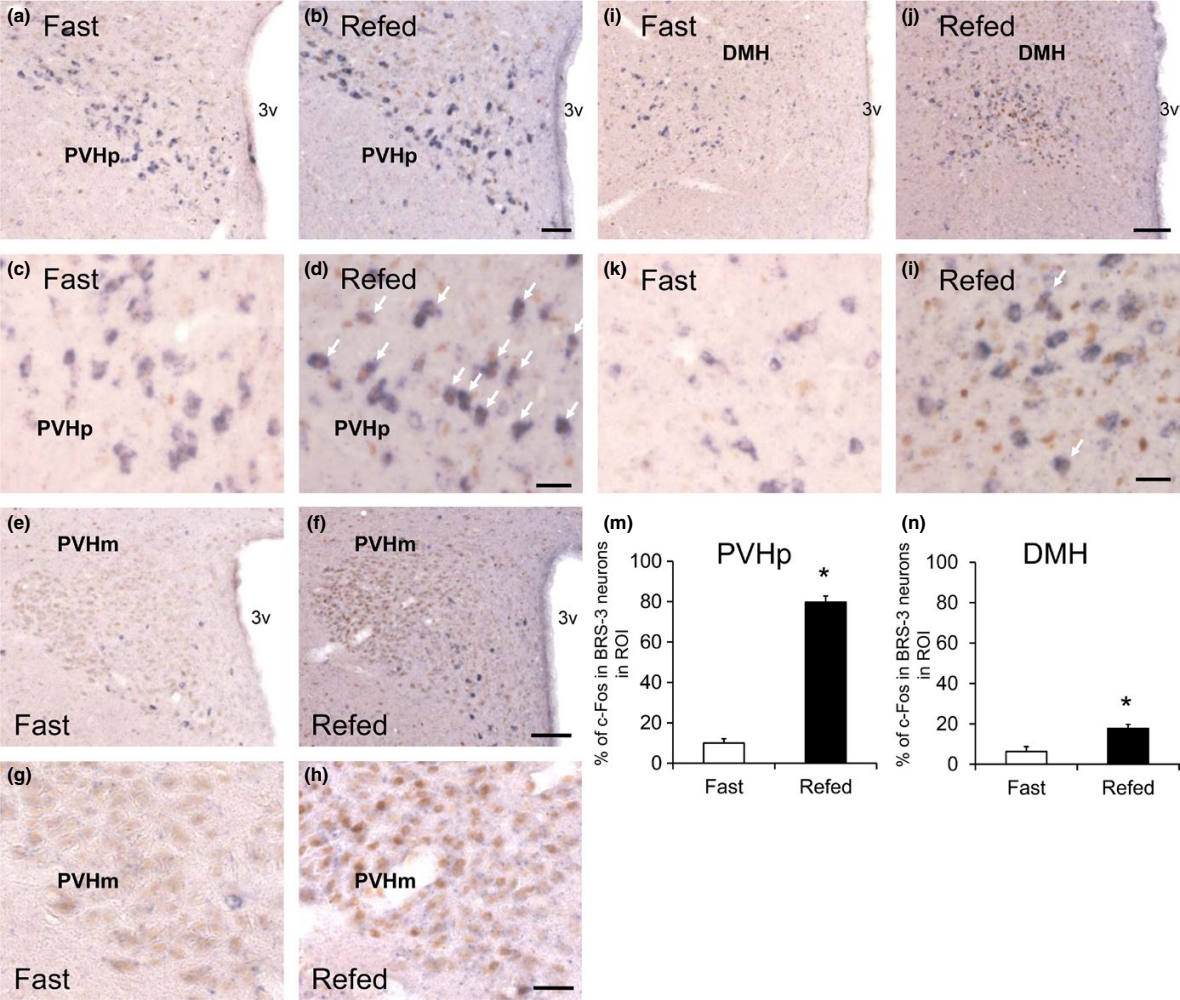
c‐Fos induction in the BRS‐3 mRNA‐positive neurons of rats refed for 2 after 48 h of food deprivation. (a–l) Coronal brain sections from rats fasted for 48 hr (Fast) or refed for 2 hr (Refed) were stained with rat BRS‐3 antisense cRNA probe and anti‐c‐Fos antibody. BRS‐3 mRNA‐positive signals (purple) and c‐Fos‐ir signals (brown) were detected in the paraventricular hypothalamic nucleus (PVH)p (a–d, bregma −1.9 mm), PVHm (e–h, bregma −1.7 mm), and dorsomedial hypothalamic nucleus (DMH) (i–l, bregma −3.2 mm). c and d, g and h, and k and l show high magnification of PVHp (a and b), PVHm (e and f), and DMH (i and j), respectively. The white arrows show double‐positive cells for BRS‐3 mRNA and c‐Fos‐ir signals. Bar: 50 μm (a and b), 25 μm (c, d, g, h, k, and l), and 100 μm (e, f, i, and j). 3v, 3rd ventricle. (m–n) The percentage of c‐Fos‐ir signals in the BRS‐3 mRNA‐positive neurons (percentage of c‐Fos in the BRS‐3 neurons in ROI) within the PVHp (m) and DMH (n) was calculated from the images; 114.3 ± 19.1 (PVHp) and 100.3 ± 10.3 (DMH) of BRS‐3 neurons in each animal were examined. Results are presented as mean values ± standard deviation (*n* = 3). *p < .05 versus vehicle (Student's *t* test)

Refeeding‐induced satiety is considered to activate anorexigenic neurons. To clarify the BRS‐3‐expressing neurons involved in feeding suppression, adult SD rats were subjected to a 48‐hr fasting; at 2 hr after refeeding, the distribution of c‐Fos‐ir neurons in the hypothalamus was evaluated using IHC. In our experimental condition, a clear and significant c‐Fos induction was mainly observed in the PVH (Figure 5a–c) and DMH (Figure 5d–f), although no c‐Fos induction was detected in the MPA (Figure 5g and h) and ARC (Figure 5i and j). Thereafter, we conducted double‐labeled staining for ISH of BRS‐3 and IHC of c‐Fos in the PVH and DMH to determine c‐Fos induction in BRS‐3‐expressing neurons. c‐Fos induction after refeeding was detected in both the parvicellular part (PVHp) and magnocellular part (PVHm) of the PVH (Figure 6a–h). BRS‐3 mRNA‐positive neurons were mainly localized in the ventral part of the PVHp, and the percentage of c‐Fos‐ir signals in the BRS‐3 mRNA‐positive neurons (percentage of c‐Fos in the BRS‐3 neurons in ROI) was significantly greater after refeeding (Figure 6a–d and m). BRS‐3 mRNA‐positive neurons were rarely observed in the PVHm (Figure 6e–h). In the DMH, clear c‐Fos induction was observed after refeeding (Figure 5d–f); however, most of these c‐Fos‐ir signals did not show colocalization with BRS‐3 mRNA‐positive neurons (Figure 6i–l). Although the c‐Fos‐ir signals in the BRS‐3 mRNA‐positive neurons were significantly increased, the percentage of c‐Fos in the BRS‐3 neurons after refeeding was only 17% (Figure 6n).

### 3.6 Induction of c‐Fos proteins in BRS‐3 mRNA‐positive neurons following single oral administration of compound‐A in DIO‐F344 rats

**Figure 7:**
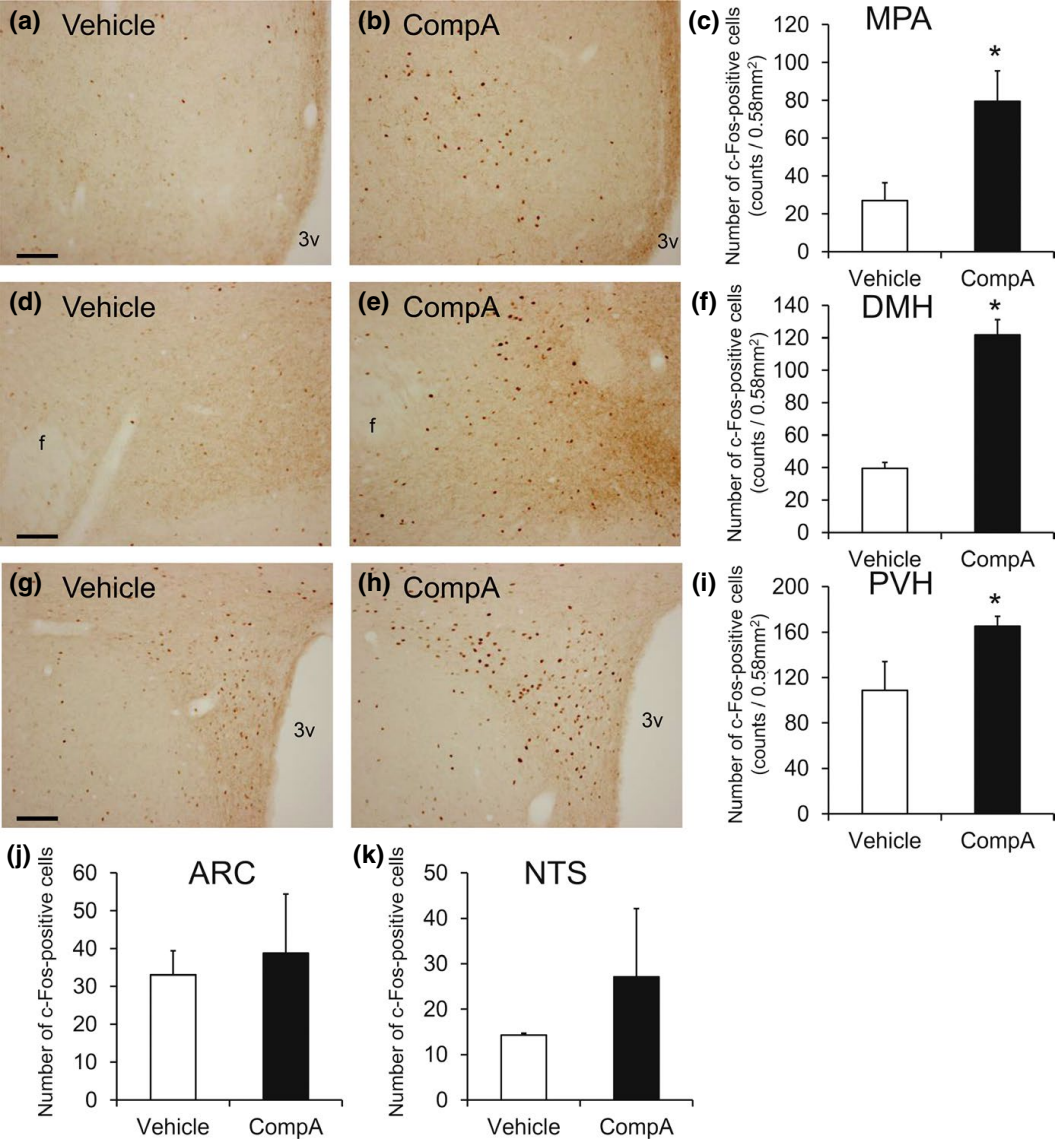
c‐Fos induction in the rats administered compound‐A. Rat brains were sampled at 2 hr after the oral administration of vehicle or 30 mg/kg of compound‐A (CompA), and the coronal sections were stained with anti‐c‐Fos antibody. (a–i) c‐Fos induction in the medial preoptic area (MPA) (a–c), dorsomedial hypothalamic nucleus (DMH) (d–f), and paraventricular hypothalamic nucleus (PVH) (g–i) following compound‐A administration. a, b, d, e, g, and h show representative photographs of c‐Fos‐ir signals in MPA (a and b, bregma −0.3 mm), DMH (d and e, bregma −3.2 mm), and PVH (g and h, bregma −1.9 mm). Bar: 100 μm. 3v, 3rd ventricle. c, f, and i show the calculated density of c‐Fos‐ir cells in the 0.58‐mm^2^ area. Average number of the total c‐Fos‐ir cells in each animal was follows: 108 ± 38 (MPA, Vehicle), 318 ± 65 (MPA, CompA), 158 ± 15 (DMH, Vehicle), 487 ± 38 (DMH, CompA), 435 ± 100 (PVH, Vehicle), and 660 ± 36 (PVH, CompA). Results are shown as mean values ± standard deviation (*n* = 4). *p < .05 versus vehicle (Student's *t* test). (j–k) Calculated density of c‐Fos‐ir cells in the 0.58‐mm^2^ area of the ARC (j) and the NTS (k). Average number of the total c‐Fos‐ir cells in each animal was follows; 132 ± 25 (ARC, Vehicle), 155 ± 63 (ARC, CompA), 57 ± 1 (NTS, Vehicle), and 109 ± 60 (PVH, CompA). Results are presented as mean values ± standard deviation (*n* = 4). *p < .05 versus vehicle (Student's *t* test)

**Figure 8:**
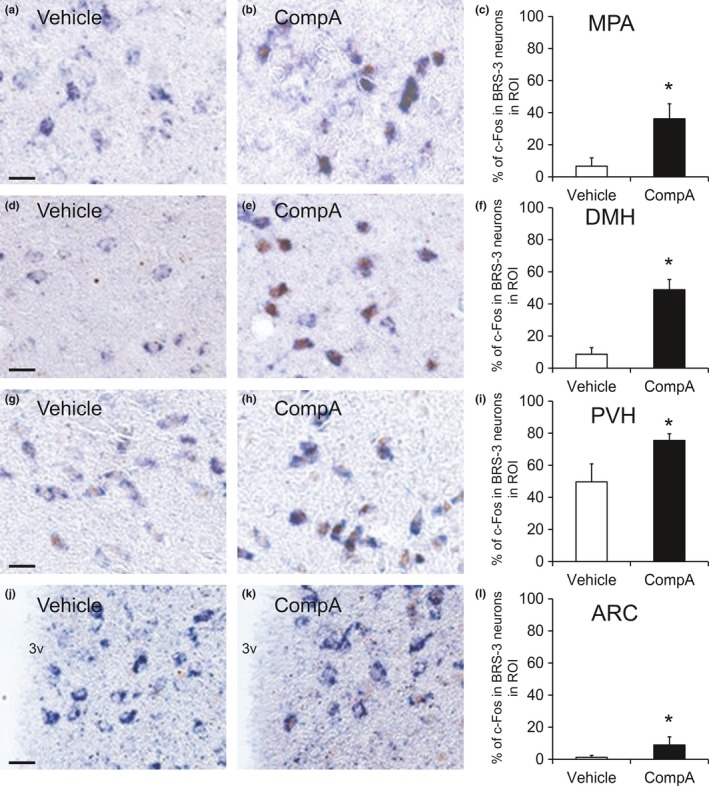
c‐Fos induction in the BRS‐3 mRNA‐positive neurons of the rats administered compound‐A. (a–l) Rat brains were sampled at 2 hr after the oral administration of vehicle or 30 mg/kg of compound‐A (CompA), and the coronal sections were stained with rat BRS‐3 antisense cRNA probe and anti‐c‐Fos antibody. BRS‐3 mRNA‐positive signals (purple) and c‐Fos‐ir signals (brown) were detected in the medial preoptic area (MPA) (a and b, bregma −0.3 mm), dorsomedial hypothalamic nucleus (DMH) (d and e, bregma −3.2 mm), paraventricular hypothalamic nucleus (PVH) (g and h, bregma −1.9 mm), and ARC (j and k, bregma −3.6 mm). Bar: 25 μm. 3v, 3rd ventricle. The percentage of c‐Fos‐ir signals in BRS‐3 mRNA‐positive neurons (percentage of c‐Fos in the BRS‐3 neurons in ROI) within the MPA (c), DMH (f), PVH (i), and ARC (l) was calculated from the images; 226.5 ± 62.2 (PVH), 243.6 ± 103.1 (DMH), 208.6 ± 57.8 (PVH), and 290.9.3 ± 87.4 (DMH) of BRS‐3 neurons in each animal were examined. Results are presented as mean values ± standard deviation (*n* = 4). *p < .05 versus vehicle (Student's *t* test)

To determine the neurons activated by BRS‐3 agonists, 30 mg/kg of compound‐A was orally administrated to DIO‐F344 rats, and the distribution of c‐Fos‐ir neurons was examined by IHC after 2 hr. The compound‐A significantly increased the c‐Fos‐ir signals in the MPA, DMH, and PVH (Figure 7a–i), although no significant increase in ARC and NTS was detected (Figure 7j and k). Hence, we performed double staining for ISH of BRS‐3 and IHC of c‐Fos in the MPA, DMH, and PVH to determine c‐Fos induction among the BRS‐3‐expressing neurons. Accordingly, the percentage of c‐Fos in the BRS‐3 neurons clearly increased in the MPA and DMH after compound‐A administration, despite the fact that these regions were barely activated by refeeding (Figure 8a–f). In the PVH, a larger percentage of c‐Fos in the BRS‐3 neurons was observed in vehicle‐treated rats (Figure 8g–i), compared with that in fasted rats (Figure 6a–d and m); this finding may be due to the ad libitum feeding of the animals. Nevertheless, a significant increase in the percentage of c‐Fos‐positive BRS‐3 neurons was observed after compound‐A administration (Figure 8g–i). In the ARC, a significant increase in the c‐Fos‐ir signals was not observed following compound‐A administration (Figure 7j). However, the percentage of c‐Fos in the BRS‐3 neurons was slightly, but significantly, increased after compound‐A administration (Figure 8j–l).

### 3.7 Anorexigenic effect of BRS‐3 agonist in Mchr1^−/−^ mice

**Figure 9:**
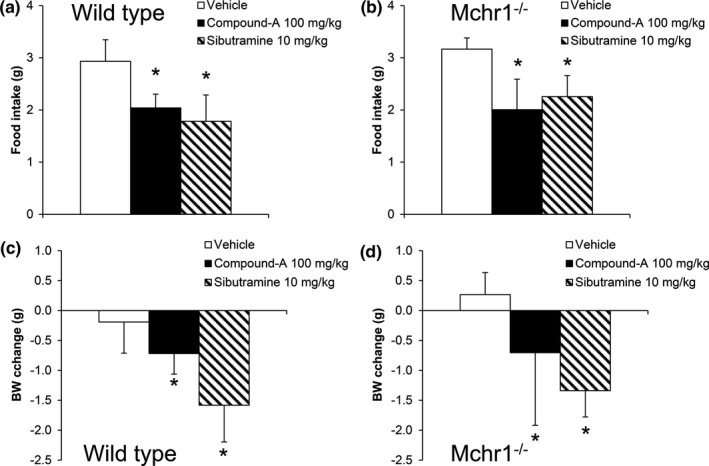
Effect of single oral administration of compound‐A on food intake and body weight in HFD‐fed wild‐type mice and HFD‐fed Mchr1^−/−^ mice. Mice were orally administered vehicle (0.5% MC solution), compound‐A (100 mg/kg), or sibutramine (10 mg/kg). (a–b) Food intake at 16 hr after dosing in HFD‐fed wild‐type mice (a) or DIO‐Mchr1^−/−^ mice (b). (c–d) Body weight change at 24 hr after dosing in HFD‐fed wild‐type mice (c) or HFD‐fed Mchr1^−/−^ mice (d). Results are shown as mean values ± standard deviation (*n* = 8). *p < .05 versus vehicle (Student's *t* test)

A previous study showed that expression of MCH and its receptor mRNA was increased in the hypothalamus of BRS‐3^‐/Y^ mice (Maekawa, Quah, Tanaka, & Ohki‐Hamazaki, 2004). Thus, the anti‐obesity effect of BRS‐3 agonist was considered to be mediated via the MCH pathway. As we have shown the anti‐obesity effect of compound‐A in mice (Nio et al., 2017), compound‐A can be used for mouse studies. Then, we attempted to validate this hypothesis using HFD‐fed Mchr1^−/−^ mice and HFD‐fed wild‐type mice. In addition to sibutramine (10 mg/kg), single oral administration of compound‐A (100 mg/kg) significantly decreased the FI and BW in both wild‐type mice and Mchr1−/− mice (Figure 9a–d).

### 3.8 Effect of compound‐A on feeding‐relating peptide gene expression in the brain of DIO‐F344 rats

**Figure 10:**
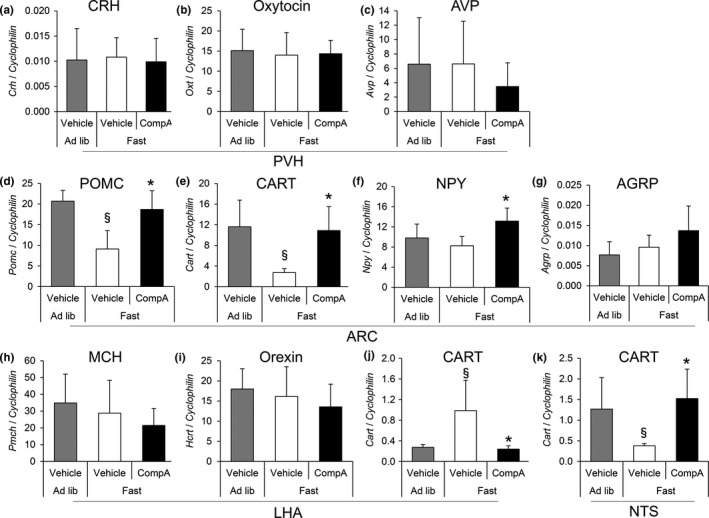
Effect of compound‐A on feeding‐relating peptide gene expression in the brain of DIO‐F344 rats. DIO‐F344 rats under ad libitum (Ad lib) conditions were fasted for 16 hr (Fast) and then orally administered with vehicle or compound‐A (30 mg/kg). At 1 hr after dosing, the brain samples were dissected and mRNA level listed in Table 2 was measured. (a–k) The expression levels of *Crh* (a), *Oxt* (b), and *Avp* (c) in the PVH,* Pomc* (d), *Cart* (e), *Npy* (f), and *Agrp* (g) in ARC,* Pmch* (h), *Hcrt* (i), and *Cart* (j) in the LHA, and *Cart* (k) mRNA in the NTS were measured via quantitative RT‐PCR. The mRNA expression of each gene was normalized to that of cyclophilin. Results are presented as mean values ± standard deviation (*n* = 5–6). ^§^ *p* < .05 versus vehicle (Ad lib), *p < .05 versus vehicle (Fast) (Student's *t* test)

To clarify the pathophysiological role of BRS‐3 on the FI in the rat brain, we measured the gene expression of feeding‐relating peptides *Pomc, Cart, Npy*,* Agrp*,* Hcrt*,* Pmch*,* Crh*,* Oxt*,* Avp*, and *Bdnf* (listed in Table 2) in the ARC, PVH, LHA, and NTS after compound‐A administration. As compound‐C did not decrease the FI and BW in normal chow‐fed SD rats (Figure 1a and b), we examined the gene expression in DIO‐F344 rats. The 16‐hr fasting condition significantly decreased *Pomc* and *Cart* mRNA expression in the ARC and *Cart* mRNA expression in the NTS (Figure 10d,e, and k), and significantly increased *Cart* mRNA expression in the LHA (Figure 10j). The single administration of compound‐A (30 mg/kg) after 16‐hr fasting significantly increased *Pomc*,* Cart,* and *Npy* mRNA expression in the ARC and *Cart* mRNA expression in the NTS (Figure 10d–f and k), and significantly decreased *Cart* mRNA expression in the LHA (Figure 10j), in comparison with the vehicle (control) in the fasting condition. The expression of *Crh*,* Oxt*, and *Avp* in PVH, *Agrp* in ARC, and *Pmch* and *Hcrt* in LHA did not show any significant change (Figure 10a–c,g–i).

## 4 DISCUSSION

In this study, we examined the hypothalamic neuronal pathway underlying energy homeostasis regulation by BRS‐3‐expressing neurons using compound‐A as a tool compound. Compound‐A is a novel orally available, selective, small‐molecule BRS‐3 agonist that we have recently reported (Nio et al., 2017). The in vivo specificity of compound‐A to BRS‐3 has been shown using BRS‐3‐deficient mice (Nio et al., 2017). In addition to our previous study, this study showed that the single oral administration of compound‐A reduced the FI and BW in DIO‐F344 rats and increased the EE in fasted DIO‐F344 rats (Figures 1 and 2). Thus, this compound can be used for analyzing the mechanism of BRS‐3‐expressing neuron‐mediated energy homeostasis under single oral administration.

Strong BRS‐3 mRNA signals were observed in the PVH, DMH, and MPA in the hypothalamus (Figure 3) (Jennings et al., 2003; Liu et al., 2002; Sano et al., 2004; Zhang et al., 2013). Hence, we focused on the neurons in these nuclei. *c‐Fos,* an immediate early gene, was used to map the neural activation following stimuli (Sakuma et al., 2015). Under our experimental conditions, refeeding was found to activate the neurons in the PVH and DMH, consistent with previous studies (Figure 5) (Angeles‐Castellanos, Aguilar‐Roblero, & Escobar, 2004; Timofeeva, Baraboi, & Richard, 2005; Timofeeva, Picard, Duclos, Deshaies, & Richard, 2002; Wu et al., 2014; Zséli et al., 2016). These data suggest that neurons in the PVH and DMH are critical for satiety regulation.

The PVH plays an important role in the regulation of numerous physiological processes, including feeding and energy metabolism (Seoane‐Collazo et al., 2015; van Swieten et al., 2014) and is roughly divided into two parts: PVHm and PVHp. The neurons in the PVHm mainly contain oxytocin and AVP, whereas those in the PVHp mainly contain CRH and TRH; these peptides suppress feeding behavior. TRH neurons in PVH are also reported to have orexigenic function (Krashes et al., 2014). Previous reports have indicated that refeeding mainly activates the AVP‐positive neurons in the PVHm and CRH‐positive neurons in the PVHp (Timofeeva et al., 2005). However, we found that the BRS‐3‐expressing neurons in the PVH did not contain CRH, oxytocin, or AVP (Figure 4). Although this is different from the previous reports that only a few CRH‐ and oxytocin‐positive neurons overlap with BRS‐3 (Bagnol & Grottick, 2008; Zhang et al., 2013), it is obvious that most of the BRS‐3 neurons in PVH do not contain CRH and oxytocin. Nevertheless, refeeding activated ~80% of the BRS‐3‐expressing neurons in the PVHp (Figure 6) and these neurons were activated by compound‐A at the dose showing anorexigenic efficacy (Figure 8). These data suggest that BRS‐3‐expressing neurons in the PVH are mainly involved in the anorexigenic action of compound‐A. Furthermore, the cluster of neurons expressing BRS‐3 was localized ventrally to the normal distribution of typical neuropeptide‐containing neurons in the PVH (Figure 4f). Thus, the cluster of BRS‐3‐expressing neurons in the PVHp could represent a novel subdivision in the PVH that plays an important role in feeding behavior through an unknown mechanism. Recently, MC4R‐expressing glutamatergic neurons in the PVH were reported to regulate feeding without any colocalization with oxytocin, CRH, AVP, or prodynorphin (Balthasar et al., 2005; Garfield et al., 2015; Shah et al., 2014). BRS‐3 expression in this neural population should be revealed in the future. Moreover, compound‐A elicits c‐Fos induction in non‐BRS‐3‐expressing neurons in the PVH (data not shown), which suggests that BRS‐3 agonists could indirectly activate these PVH neurons. This indirect PVH activation can be mediated through BRS‐3‐expressing neurons in other nuclei (such as in the LPB) that project to the PVH (Zséli et al., 2016).

Refeeding activated the neurons in the DMH (Figure 5), but the number of refeeding‐activated BRS‐3‐expressing neurons was ~20% (Figure 6). Hence, most of the BRS‐3‐expressing neurons in the DMH are not involved in refeeding‐induced satiety. The DMH is known to be closely involved in various functions, including feeding behavior, thermoregulation, circadian rhythm, cardiovascular function, locomotor activity, stress, and reproduction (Lo et al., 2016; Morrison, 2016; van Swieten et al., 2014). The regulatory role of the DMH in energy homeostasis is well established in lesion studies, and leptin is known to directly activate neurons in the DMH (Bellinger & Bernardis, 2002; van Swieten et al., 2014). Recently, Garfield et al. reported that leptin receptor‐expressing GABAergic DMH neurons (ventral compartment) play an important role for suppression of ARC AgRP neurons (Garfield et al., 2016). The refeeding‐sensitive BRS‐3‐expressing neurons in the DMH might belong to this feeding‐relating neuronal cluster. Furthermore, compound‐A activated approximately 50% of BRS‐3‐expressing neurons in the DMH (Figure 8). Although compound‐A may directly activate these DMH neurons, its anorexigenic effect may not be as profound as compared to the other functions, including EE enhancement.

The BRS‐3‐expressing neurons in the MPA were not sensitive to refeeding (Figure 5), but were clearly activated by compound‐A (Figure 8). GABAergic neurons in the MPA are known to play central roles in thermoregulation (Morrison, 2016). Thermal sensory inputs are integrated in the preoptic area, including the MPA and MnPO, and are transmitted to the DMH, rostral raphe pallidus, and preganglionic sympathetic neurons. Previous reports have shown that BRS‐3 agonist regulates body temperature, blood pressure, and heart rate via a central sympathetic mechanism (Lateef et al., 2014, 2016; Metzger et al., 2010). As Bagnol et al. have reported that ~80% of BRS‐3‐expressing neurons in the MPA and ventromedial preoptic nucleus are GABAergic (Bagnol & Grottick, 2008), the BRS‐3‐expressing neurons in the MPA could play an important role in energy metabolism through the sympathetic nerve. Intriguingly, neurons in the DMH are known to mediate the MPA‐induced thermogenic regulation in brown adipose tissue and are considered as thermogenesis‐promoting neurons (Morrison, 2016). Hence, the activation of BRS‐3 neurons in the DMH by compound‐A could lead to peripheral EE enhancement through this sympathetic pathway. However, the thermogenesis‐promoting neurons in the DMH are inhibited by GABAergic neurons in the MPA. Thus, it is less likely that the BRS‐3‐expressing GABAergic neurons in the MPA project to the DMH. One possibility is that BRS‐3 is expressed in GABAergic interneurons in the MPA or MnPO, which inhibit the neurons in the MPA that project to the DMH. Hence, the regulatory mechanism underlying EE in BRS‐3 neurons needs to be investigated further.

The ARC plays a key role in energy homeostasis (Seoane‐Collazo et al., 2015; van Swieten et al., 2014). It possesses orexigenic NPY/AgRP neurons and anorexigenic POMC/CART neurons. However, BRS‐3‐expressing neurons in the ARC did not exhibit *Npy* or *Pomc* mRNA signals (Figure 4), consistent with previous studies (Zhang et al., 2013). In our experimental condition, refeeding did not induce c‐Fos expression in the ARC (Figure 5) and compound‐A administration induced only slight c‐Fos induction in BRS‐3‐expressing neurons (Figure 8). As previous study showed that refeeding significantly leads to c‐Fos induction in ARC (Wu et al., 2014), the lack of c‐Fos induction in this study was due to the difference of experimental condition. In addition, it would be supposed that limited c‐Fos induction by compound‐A might be due to potent inhibitory regulation to these neurons. Hence, the involvement of these BRS‐3‐expressing neurons in energy homeostasis remains unknown. Nonetheless, we found that the expression of *Pomc, Cart, and Npy* mRNA is increased in the ARC at 1 hr after compound‐A administration (Figure 9). This suggests that there are indirect pathways to activate these neurons using BRS‐3 agonists. Although compound‐A did not elicit significant c‐Fos induction, the percentage of c‐Fos in the BRS‐3 neurons was slightly, but significantly, increased in the ARC (Figure 8). Recently, Kong et al. reported on the presence of GABAergic neurons in the ARC, without NPY, AgRP, POMC, or CART expression, that regulate EE (Kong et al., 2012). Moreover, Bagnol et al. reported that approximately 80% of the BRS‐3‐expressing neurons in the posterior ARC are GABAergic (Bagnol & Grottick, 2008). Thus, it is likely that BRS‐3 neurons in the ARC may be involved in EE enhancement.

Although we focused on hypothalamic regulation by the BRS‐3 agonist, there are several extrahypothalamic nuclei where BRS‐3 is strongly expressed, such as in the LPB and MHb (Figure 3). LPB reportedly works as a hub that integrates signals from several brain regions to modulate feeding and BW (Wu, Boyle, & Palmiter, 2009; Wu, Clark, & Palmiter, 2012). LPB also reportedly mediates the transmission signals from cutaneous thermoreceptors in thermoregulation (Morrison, 2016). Furthermore, MHb plays an important role in stress, memory, and nicotine withdrawal, and has a neural connection with the LHb that is related to the reward and aversion functions (Meye, Lecca, Valentinova, & Mameli, 2013; Viswanath, Carter, Baldwin, Molfese, & Salas, 2013). Thus, LPB and MHb could be involved in the anti‐obesity effect of the BRS‐3 agonist. Nevertheless, further study on the extrahypothalamic regulation by BRS‐3 agonist is needed.

The anti‐obesity effect of the BRS‐3 agonist was still reportedly observed in Npy−/−, Mc4r−/−, Cb1r−/−, and Lepr−/− mice (Guan et al., 2010). Moreover, we found that the anti‐obesity effect by compound‐A was not changed in Mchr1^−/−^ mice (Figure 9) and MCH expression was unchanged after compound‐A administration (Figure 10). Bagnol. et al. has reported that no BRS‐3 mRNA expression is observed in the LHA MCH neurons (Bagnol & Grottick, 2008), and thus, it is likely that MCH pathway is not important for BRS‐3‐mediated energy regulation. These results strongly support our hypothesis that the well‐known feeding‐related peptides are not the primary regulators involved in the anti‐obesity effect mediated by the BRS‐3‐expressing neurons. On the other hand, we found a significant change in the expression of *Pomc* mRNA in the ARC and *Cart* mRNA in the ARC, LHA, and NTS as a result of compound‐A administration (Figure 10). Hence, these peptides could lead to at least partial energy regulation by BRS‐3, whereas we observed a compound‐A‐induced increase of *Npy* mRNA, a potent orexigenic peptide, in ARC (Figure 10). As it is clear that BRS‐3 agonist finally exerts anti‐obesity effects, this NPY increase might be a compensatory reaction. Regarding this controversy, further investigation is needed.

This study has certain limitations. As only acute responses in the CNS were examined, we cannot exclude the possibility that the chronic responses of BRS‐3‐expressing neurons associated with energy homeostasis would differ from our results. Moreover, even in the hypothalamus, there are areas that have not been examined, such as BRS‐3‐expressing neurons in the SCN. SCN may be involved in the BRS‐3 agonist‐mediated anti‐obesity effect via circadian rhythm regulation (Nio et al., 2017). The histological assessment in this study was conducted under nonbiased condition, although the quantification method does not fully satisfy the criteria of “Stereological methods” stated in Schmitz & Hof, 2005. In detail, the ROIs in this study were not identified with the Nissl staining of adjacent sections and were not fully covered with the regularly spaced series of sections. Strict stereological method should have been applied; however, the ROIs in this study were reliably identified using the standard cytoarchitectonic and anatomical landmarks as reported in previous studies (Göktalay & Millington, 2016; Ryan et al., 2014), and the procedures including the section selection were conducted under blinded conditions.

In conclusion, the present study supposed that BRS‐3‐expressing neurons regulate energy homeostasis through a novel neuronal pathway in the hypothalamus. Our findings suggested that BRS‐3 could be used as a marker of a novel neuronal population associated with energy homeostasis. Still, our hypothesis should be confirmed through more direct methodology, such as optogenetics and DREADD (Designer Receptors Exclusively Activated by Designer Drugs) in the future research. At present, no high‐affinity endogenous ligand of BRS‐3 has been identified, except for that of the BRS‐3 phylogenetic subgroup in *Drosophila* (Ida et al., 2012; Ikeda et al., 2015; Sano et al., 2015). Further study of the BRS‐3‐related neural pathway, including the deorphanization of BRS‐3, is needed to determine the mechanism underlying energy homeostasis and to evaluate BRS‐3 as a potential therapeutic target.

## CONFLICT OF INTERESTS

The authors declare no competing financial interests.

## AUTHOR CONTRIBUTION

M. Maruyama, NH, and Y. Nio conceived and designed the experiments; M. Maruyama, NH, Y. Nio, KH, TN, MF, JS, MN, and NA performed the experiments and analyzed data; MN, TO, YA, SS, and SK contributed reagents and materials; M Maruyama wrote the manuscript; Y. Nagisa, YH, and M. Mori contributed to experimental design; M. Maruyama, NH, and Y. Nio should be considered joint first author.
